# Characterization of Induced Pluripotent Stem Cell-derived Human Serotonergic Neurons

**DOI:** 10.3389/fncel.2017.00131

**Published:** 2017-05-08

**Authors:** Lining Cao, Rui Hu, Ting Xu, Zhen-Ning Zhang, Weida Li, Jianfeng Lu

**Affiliations:** ^1^Translational Medical Center for Stem Cell Therapy and Institute for Regenerative Medicine, Shanghai East Hospital, School of Life Sciences and Technology, Tongji UniversityShanghai, China; ^2^Waisman Center, University of WisconsinMadison, WI, USA

**Keywords:** serotonergic neuron, induced pluripotent stem cell, neurotoxin, transplantation, raphe nucleus

## Abstract

In the brain, the serotonergic neurons located in the raphe nucleus are the unique resource of the neurotransmitter serotonin, which plays a pivotal role in the regulation of brain development and functions. Dysfunction of the serotonin system is present in many psychiatric disorders. Lack of *in vitro* functional human model limits the understanding of human central serotonergic system and its related diseases and clinical applications. Previously, we have developed a method generating human serotonergic neurons from induced pluripotent stem cells (iPSCs). In this study, we analyzed the features of these human iPSCs-derived serotonergic neurons both *in vitro* and *in vivo*. We found that these human serotonergic neurons are sensitive to the selective neurotoxin 5, 7-Dihydroxytryptamine (5,7-DHT) *in vitro*. After being transplanted into newborn mice, the cells not only expressed their typical molecular markers, but also showed the migration and projection to the host’s cerebellum, hindbrain and spinal cord. The data demonstrate that these human iPSCs-derived neurons exhibit the typical features as the serotonergic neurons in the brain, which provides a solid foundation for studying on human serotonin system and its related disorders.

## Introduction

In the brain, serotonergic neurons are located in the raphe nucleus. Although there are only about 300,000 serotonergic neurons in the human brain (Chen and Condron, 2008), they innervate the whole central nervous system, with the rostral group (B5-B9 subgroups) mainly projecting to the brain and the caudal group (B1-B4 subgroups) primarily sending axons to the spinal cord (Goridis and Rohrer, 2002). Since they are the primary source and a site for re-uptake of serotonin in the brain, serotonergic neurons are essential for a series of serotonin-related neural functions (including regulating mood, affection, cognition, aggression, satiety, sleep and numerous other autonomic functions) and are a major therapeutic target for a wide spectrum of serotonin dysfunction-related psychiatric disorders (such as schizophrenia, depression, bipolar disorder, anxiety, obsessive-compulsive disorder, chronic pain syndrome and eating disorders; Bethea et al., 2002; Wichers and Maes, 2002; Donati and Rasenick, 2003). Establishment of *in vitro* human serotonergic neurons model system will help the understanding of their roles in brain physiology and in the development of diseases.

Deep in the brain, it is difficult to obtain human serotonergic neurons via biopsy and primary culture. Embryonic stem cells (ESCs) and induced pluripotent stem cells (iPSCs) make it possible to establish an *in vitro* system to investigate serotonergic neurons. Differentiation of serotonergic neurons has been attempted using mouse, monkey and human ESCs. During dopamine neurons differentiation from mouse ESCs in the presence of fibroblast growth factors (FGF2, FGF4 and/or FGF8), serotonergic neurons, based on staining for serotonin, have been observed (Lee et al., 2000; Kim et al., 2002; Barberi et al., 2003). Similarly, monkey ESCs have been differentiated to serotonergic neurons in the presence of FGF2 and/or FGF4, and the proportion of serotonin-expressing cells is reported high (Salli et al., 2004; Tokuyama et al., 2010). In a report on serotonergic neuron differentiation from human ESCs, a similar strategy (with the treatment of FGF1, FGF2, retinoic acids and serotonin) is employed for differentiation but the efficiency is very low (Kumar et al., 2009).

Recently, we have demonstrated the accurate timely regulation of WNT, SHH and FGF4 signaling pathways during the serotonergic neuron differentiation and generated an enriched population of serotonergic neurons from human ESCs and iPSCs (Lu et al., 2016). These human serotonergic neurons not only express bio-markers (TPH2, serotonin, GATA3, GATA2, FEV, LMX1B, SERT, AADC and VMAT2; Deneris and Wyler, 2012), but also show electrophysiological activities and release serotonin in response to stimuli, including FDA-approved drugs, in a dose- and time-dependent manner.

In this study, we further analyzed the features of human iPSCs-derived serotonergic neurons both *in vitro* and *in vivo*. We found that these human serotonergic neurons are sensitive to the specific neurotoxin 5,7-Dihydroxytryptamine (5,7-DHT) *in vitro*. After being transplanted into newborn mice, the cells not only expressed their typical molecular markers, but also showed the migration and projection to the cerebellum, hindbrain and spinal cord. The data demonstrate that these human iPSCs-derived neurons exhibit the typical features as the serotonergic neurons in the brain, which provides a solid foundation for studying on human serotonin system and its related disorders.

## Materials and Methods

### Human iPSCs Culture

Human iPSCs (GM15, passages 35–50, home-made cell line) were maintained on mouse embryonic fibroblast (MEF) feeder in a stem cell growth medium (Hu et al., 2010). The human iPSCs were established using retroviral approaches with Yamanaka factors (Takahashi et al., 2007).

### Human Serotonergic Neuron Differentiation

The process for human serotonergic neuron generation is described previously (Lu et al., 2016). Briefly, human iPSCs were seeded onto Laminin (Life Technologies)-coated plastic plates or polyornithine (PO, from Sigma-Aldrich) and Laminin-coated 12 mm glass coverslips. iPSCs at approximately 20% confluence (1 day after passing) were cultured for 1 week in a chemically defined medium (CDM) modified from our previous work (Lu et al., 2013). Briefly, the medium consists of DMEM/F12: Neurobasal (1:1), 1× N2, 1× B27, 1× nonessential amino acids (NEAA), 1% GlutaMAX (all from Life Technologies), 2 μM SB431542 (Stemgent), 2 μM DMH1 (Tocris Bioscience) and 1.4 μM CHIR99021. On the second week of differentiation, cells were passed mechanically in the same CDM at the ratio of 1:3 onto pre-coated plates or coverslips and SHH C25II (1000 ng/ml, R&D Systems) were applied to ventralize cells. On the third week of differentiation, cells were passed as previously in the same CDM with 1000 ng/ml SHH, and FGF4 (10 ng/ml) was used to help the specification of the serotonergic fate. From the fourth week, serotonergic progenitors were seeded onto PO- and Laminin-coated glass coverslips and cultured in a neuronal differentiation medium (NDM) consisting of Neurobasal with 1× N2, 1× B27, 1× NEAA supplemented with 1 μg/ml laminin, 0.2 mM vitamin C (Tocris Bioscience), 2.5 μM DAPT (Tocris Bioscience), 10 ng/ml glial cell line-derived neurotrophic factor (GDNF), 10 ng/ml brain-derived neurotrophic factor (BDNF), 10 ng/ml insulin-like growth factor-I (IGF-I) and 1 ng/ml transforming growth factor β3 (all from PeproTech). 5,7-DHT and 6-OHDA are both purchased from Sigma.

### Immunocytochemistry

Immunostaining was performed as described previously (Lu et al., 2016). In brief, cells were fixed in 4% neutral-buffered paraformaldehyde (PFA) for 20 min at room temperature. All antibodies, sources, and dilutions are listed in Supplementary Table S1. Cell populations were counted among total cells (Hoechst labeled) using the ImageJ software. Five fields of each coverslip were chosen randomly, and three coverslips in each group were counted. Data were expressed as mean ± SEM.

### Cell Transplantation and Histology

Transplantation studies were conducted following protocols approved by the Animal Care and Use Committees at the University of Wisconsin-Madison. Human iPSCs-derived serotonergic progenitors (10^5^ cells in 2 μl artificial cerebral spinal fluid, Harvard Apparatus) were injected 2 mm after lambda along the midline and 3 mm deep into the 4th ventricles of P1 newborn severe combined immunodeficiency (SCID)-beige (Taconic) mice. Three months after transplantation, animals were processed for histological analysis as described before Ma et al. (2012). All antibodies, sources and dilutions are listed in Supplementary Table S1.

### Statistical Analysis

Values were expressed as mean ± SEM. Differences between means were assessed by *t* test. A *p* value <0.05 was considered statistically significant.

## Results

### Human iPSCs-Derived Serotonergic Neurons Are Sensitive to 5,7-DHT *In Vitro*

**Figure 1:**
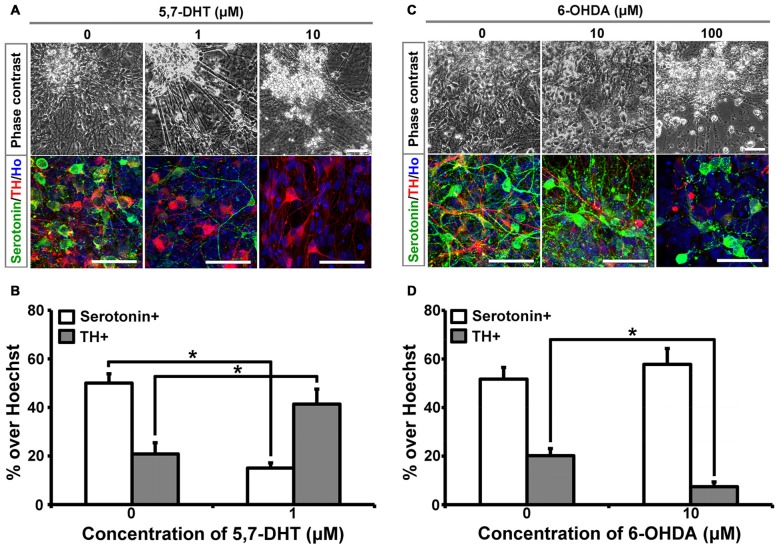
**Human induced pluripotent stem cells (iPSCs)-derived serotonergic neurons are sensitive to 5,7-DHT *in vitro*. (A)** Phase contrast images and immunostaining images of the cells treated with different concentrations of 5,7-DHT. **(B)** Quantification of Serotonin positive or TH positive cells in **(A)** (*n* = 3). Data are represented as mean ± SEM. **(C)** Phase contrast images and immunostaining images of the cells treated with different concentrations of 6-OHDA. **(D)** Quantification of Serotonin positive or TH positive cells in **(C)** (*n* = 3). Data are represented as mean ± SEM. *P < 0.05, Student’s *t*-test. Scale bar: 50 μm; Red: TH+ cells; Green: Serotonin+ cells; Blue: Hoechst staining (Ho).

5,7-DHT is a selective neurotoxin for serotonergic neurons. When different concentrations of 5,7-DHT were applied to the 6-week-old cultures for 3 days, human iPSCs-derived serotonergic neurons were selectively killed, which is indicated by the loss of serotonin+ cells in the culture (Figures 1A,B). In order to confirm the selective neurotoxic effect of 5,7-DHT on human iPSCs-derived serotonergic neurons, 6-hydroxydopamine (6-OHDA) was applied to the same culture system. Immunostaining data showed that 10 μM 6-OHDA could selectively kill the TH+ neurons and leave the serotonergic neurons alive in the culture (Figures 1C,D). It is noted that high concentration of 5,7-DHT (10 μM) and 6-OHDA (100 μM) showed non-specific effects and killed most of the cells in the culture (Figures 1A,C). This data demonstrates that human iPSCs-derived serotonergic neurons are sensitive to the selective neurotoxin 5,7-DHT as the serotonergic neurons in the brain. It is a very useful property for the *in vitro* human serotonergic neuron system, which could be applied for the selective neurotoxin screening in the future.

### Human iPSCs-Derived Serotonergic Neurons Migrate and Project to the Host’s Cerebellum, Hindbrain and Spinal Cord *In Vivo*

**Figure 2:**
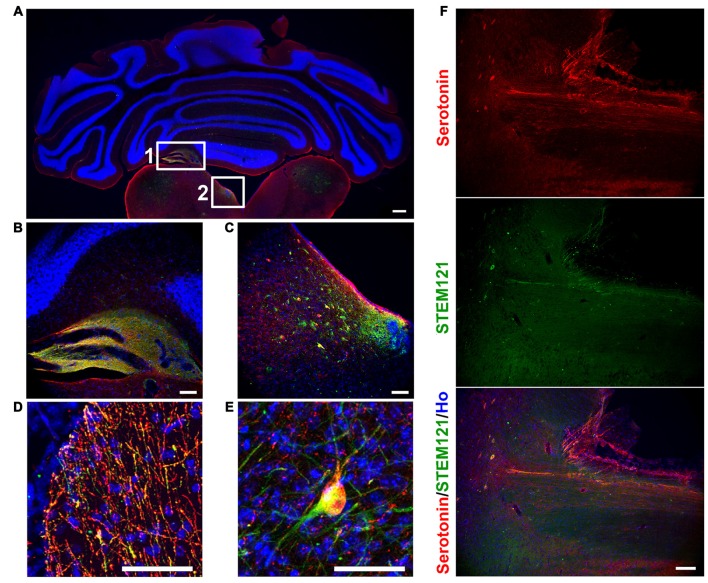
**Human iPSCs-derived serotonergic neurons migrate and project to the host’s cerebellum, hindbrain and spinal cord *in vivo*. (A–C)** After 3 months of transplantation, human serotonergic neurons formed chimera with the host’s brain tissues around the 4th brain ventricle. **(B)** is the detailed image for inset 1 in **(A)**; **(C)** is the detailed image for inset 2 in **(A)**. **(D)** Serotonin+ human fibers were observed in the host’s cerebellum. **(E)** Serotonin+ human cell bodies were observed in the host’s ventral hindbrain. **(F)** Serotonin+ human fibers were observed in the host’s spinal cord. Scale bar: **(A)** 200 μm; **(B–F)**, 50 μm; Red: Serotonin+ cells; Green: STEM121+ cells (human cells); Blue: Hoechst staining (Ho).

In order to see the *in vivo* features of human iPSCs-derived serotonergic neurons, human iPSCs-derived serotonergic progenitors were transplanted into the 4th brain ventricle of P1 newborn mice. Three months after transplantation, human grafts, which were positive for both human specific marker STEM121 and serotonin, formed chimera with the host’s tissues around the 4th brain ventricle (Figures 2A–C). Although most of the cells were still in the grafts, a substantial number of serotonin+ human cells were found in the dorsal hindbrain area (Figure 2C), while a few serotonin+ human fibers and cell bodies were observed in the cerebellum and ventral hindbrain respectively (Figures 2D,E). More interestingly, human iPSCs-derived serotonergic neurons even sent fibers to the host’s spinal cord (Figure 2F). This indicates that human iPSCs-derived serotonergic neurons could adapt to the brain environment, survive, migrate from the transplanted progenitor niches and project neurofibers to the host’s central nervous system.

### Transplanted Human Cells Exhibit the Typical Bio-Markers of Neurons

**Figure 3:**
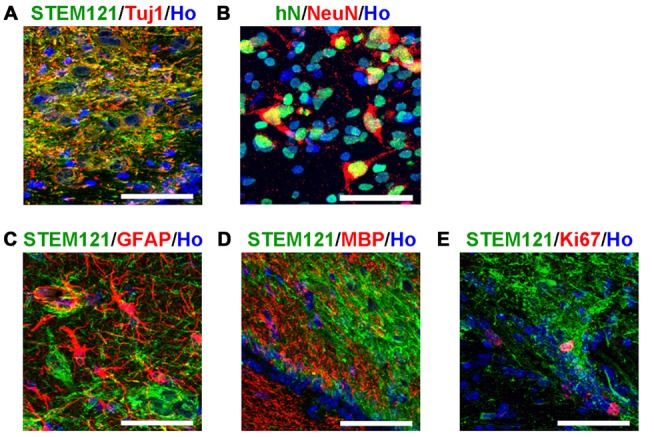
**Transplanted human cells exhibit the typical bio-markers of neurons. (A)** Transplanted human cells stained with human specific markers STEM121 (Green) and neuronal marker Tuj1 (Red). **(B)** Transplanted human cells stained with human specific markers human Nuclei (hN, Green) and neuronal marker NeuN (Red). **(C)** Transplanted human cells (STEM121+, Green) were not stained with astrocyte marker GFAP (Red). **(D)** Transplanted human cells (STEM121+, Green) were not stained with oligodendrocyte marker MBP (Red). **(E)** A few of transplanted human cells (STEM121+, Green) were stained with dividing cell marker Ki67 (Red). Scale bar: 50 μm; Blue: Hoechst staining (Ho).

In order to systematically assess the features of the transplanted cells, we first detected the transplanted human cells with pan-neural markers. The majority of the transplanted human cells (in the human grafts around the 4th brain ventricle) became neurons, indicated by Tuj1 and NeuN staining (Figures 3A,B); few astrocytes, oligodendrocytes or dividing progenitors were observed, indicated by negative GFAP, MBP or Ki67 staining respectively (Figures 3C–E). This indicates that most of the human iPSCs-derived serotonergic progenitors differentiated into neurons after transplantation.

### Transplanted Human Cells Exhibit the Typical Bio-Markers of Serotonergic Neurons

**Figure 4:**
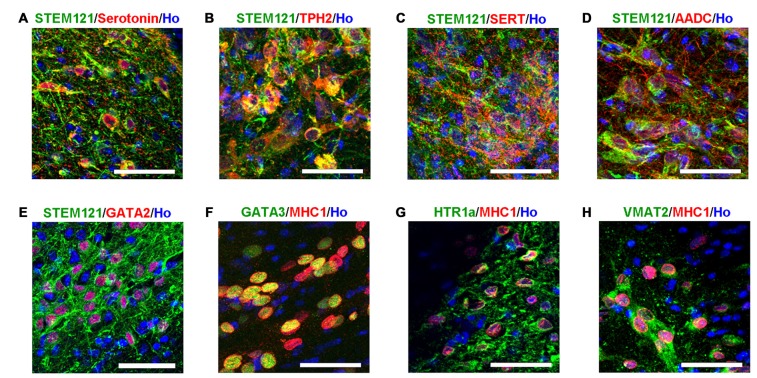
**Transplanted human cells exhibit the typical bio-markers of serotonergic neurons. (A)** Transplanted human cells (STEM121+, Green) were stained with Serotonin (Red). **(B)** Transplanted human cells (STEM121+, Green) were stained with TPH2 (Red). **(C)** Transplanted human cells (STEM121+, Green) were stained with SERT (Red). **(D)** Transplanted human cells (STEM121+, Green) were stained with AADC (Red). **(E)** Transplanted human cells (STEM121+, Green) were stained with GATA2 (Red). **(F)** Transplanted human cells (MHC1+, Red) were stained with GATA3 (Green). **(G)** Transplanted human cells (MHC1+, Red) were stained with HTR1a (Green). **(H)** Transplanted human cells (MHC1+, Red) were stained with VMAT2 (Green). Scale bar: 50 μm; Blue: Hoechst staining (Ho).

Transplanted cells (in the human grafts around the 4th brain ventricle) were further identified with bio-markers of serotonergic neurons. It was observed that the transplanted human cells (labeled by either STEM121 or human specific MHC1) expressed serotonin neuron specific marker serotonin and TPH2, as well as other markers for serotonergic neurons, such as GATA2, GATA3, AADC, HTR1a and VMAT2 (Figure 4). It is noted that SERT, a marker of mature and functional serotonergic neurons, was also detected in human iPSCs-derived serotonergic neurons after transplantation (Figure 4C), while it is very hard to be stained *in vitro* (data not shown). This data indicates that most of the transplanted cells became serotonergic neurons and the host’s brain environment help them become mature and functional.

## Discussion

In this study, we characterized the features of human iPSCs-derived serotonergic neurons both *in vitro* and *in vivo*.

As early as 17 years ago, it was reported that serotonergic neurons could be obtained from mouse ESCs (Lee et al., 2000); however, because the studies at that moment mostly focused on the effects of dopamine neurons on Parkinson’s disease (Kim et al., 2002; Barberi et al., 2003), the detailed features of ESCs-derived serotonergic neurons were not well characterized. With the advance of the knowledge and techniques for neural differentiation from rhesus monkey and human ESCs, serotonergic neurons derived from primate ESCs were generated (Salli et al., 2004; Kumar et al., 2009; Tokuyama et al., 2010). Rhesus ESCs-derived serotonergic neurons not only express bio-markers for serotonergic neurons, but also exhibit functional high-affinity transporter sites, as well as high-affinity 5HT1A binding sites, which are essential targets of common psychoactive drugs (Salli et al., 2004; Tokuyama et al., 2010). In the human ESCs-derived serotonergic neurons, the serotonin content, the localization of serotonin vesicles and their ability to release serotonin following depolarization were characterized using a live cell serotonin imaging technique based on three-photon microscopy (Kumar et al., 2009). High efficient generation of human serotonergic neurons from both ESCs and iPSCs was reported by us recently (Lu et al., 2016). The typical bio-markers and the *in vitro* electrophysiological features of serotonergic neurons were assayed on these neurons; in addition, because of the enriched population of serotonergic neurons, it makes possible to detect the serotonin neurotransmitter released in the medium via ultra-performance liquid chromatography-electrospray ionization-tandem mass spectrometry (UPLC-ESI-MS/MS), which facilitates this culture system to serve as a platform to validate serotonin enhancers and selective serotonin reuptake inhibitor (SSRIs). Although systematically studied in our previous work, some important features of serotonergic neurons still need to be validated on human iPSCs-derived serotonergic neurons.

Sensitivity to the selective neurotoxin 5,7-DHT is an important feature for serotonergic neurons. 5,7-DHT is a classical neurotoxin used to selectively kill serotonergic neurons and decrease concentrations of serotonin in the brain (Cairncross et al., 1977; Liu et al., 2007). Human iPSCs-derived serotonergic neurons were selectively killed by a proper dosage of 5,7-DHT, which mimics the response to 5,7-DHT as serotonergic neurons do in the brain. In our culture system, there are around 50% serotonergic neurons and 20%–25% TH+ neurons (Lu et al., 2016). This mixed culture system gives us a chance to confirm the specific neurotoxic effect of 5,7-DHT on human iPSCs-derived serotonergic neurons by the application of 6-OHDA, a neurotoxic synthetic organic compound used to selectively destroy TH+ dopaminergic and noradrenergic neurons in the brain (Kelly et al., 1975). As expected, 6-OHDA selectively killed TH+ neurons but left serotonergic neurons in the culture alive. This feature demonstrates that these human iPSCs-derived cells are serotonergic neurons which are sensitive to its selective neurotoxin. In addition, it is possible to use this *in vitro* culture system to screen serotonergic neuron-related neurotoxins and explore the mechanisms of how the neurotoxins damage serotonergic neurons selectively.

The *in vivo* features of human iPSCs-derived serotonergic neurons are very important but were never reported. In order to see the characterizations of maturation, migration, projection and cell-identity of human iPSCs-derived serotonergic neurons in a brain environment, human iPSCs-derived serotonergic progenitors were transplanted into the newborn mice. Since in the brain serotonergic neurons localize in the raphe nucleus of hindbrain, a region close to the 4th brain ventricle, the progenitors were injected into the 4th brain ventricle. Three months later, human grafts were found in the wall of the host’s 4th brain ventricle, and many human cells became mature neurons by expressing NeuN. Most of the transplanted cells expressed bio-markers for serotonergic neurons. More importantly, these human cells not only migrate to the areas of dorsal and ventral hindbrain, but also projected the outgrowth into cerebellum and even spinal cord. *In vitro*, the human iPSCs-derived serotonergic neurons expressed regional markers with the r2–3 region identities, which locate in the median raphe (Lu et al., 2016). Some of the serotonergic neurons in the median raphe project axons to the spinal cord (Bang et al., 2012; Alonso et al., 2013). Our transplantation data is consistent with the previous observation in the animal’s brain. Although it is reported that serotonergic neurons in the median raphe also project ascending axons to the anterior regions, we did not found human serotonin fibers in the anterior regions of the host’s brain (data not shown). It may require a longer term for observation and more careful quantification of neural fiber projection to confirm the innervation bias of human iPSCs-derived serotonergic neurons. In this study, the successful survival of the transplanted human iPSC-derived serotonergic neurons in the animal’s brain is the basis for the future functional assess. Transplantation of human iPSC-derived serotonergic cells into animal models with the deficiency of brain serotonergic neurons, such as the conditional Lmx1-b knockout mice whose central serotonergic neurons are almost completely disappeared (Hodges et al., 2008), will help the understanding of the *in vivo* function of human iPSCs-derived serotonergic neurons. Furthermore, in the future study, synapse formation is required to be detected to confirm the ability of human iPSCs-derived serotonergic neurons to form neural circuits with host’s brain.

Taken together, our study demonstrates human iPSCs-derived neurons exhibit the typical features of the serotonergic neurons in the brain, which provides a solid foundation for studying on human serotonin system and its related disorders.

## Author Contributions

LC designed and performed experiments, analyzed data and co-wrote the article. RH, TX, Z-NZ and WL cultured cells and performed Immunocytochemistry experiments. JL performed animal experiments, designed experiments and co-wrote the article.

## Funding

This work is funded by the Major Program of Development Fund for Shanghai Zhangjiang National Innovation Demonstration Zone “Translational Medicine Center for Stem Cell Therapy of Shanghai Zhangjiang National Innovation Demonstration Zone” (grant no. ZJ2014-ZD-002).

## Conflict of Interest Statement

The authors declare that the research was conducted in the absence of any commercial or financial relationships that could be construed as a potential conflict of interest. The handling Editor declared a shared affiliation, though no other collaboration with the authors and states that the process nevertheless met the standards of a fair and objective review.

## References

[B1] Alonso A. Merchan P. Sandoval J. E. Sanchez-Arrones L. Garcia-Cazorla A. Artuch R.. (2013). Development of the serotonergic cells in murine raphe nuclei and their relations with rhombomeric domains. Brain Struct. Funct. 218, 1229–1277. 10.1007/s00429-012-0456-8 23052546 PMC3748323

[B2] Bang S. J. Jensen P. Dymecki S. M. Commons K. G. (2012). Projections and interconnections of genetically defined serotonin neurons in mice. Eur. J. Neurosci. 35, 85–96. 10.1111/j.1460-9568.2011.07936.x 22151329 PMC3268345

[B3] Barberi T. Klivenyi P. Calingasan N. Y. Lee H. Kawamata H. Loonam K.. (2003). Neural subtype specification of fertilization and nuclear transfer embryonic stem cells and application in parkinsonian mice. Nat. Biotechnol. 21, 1200–1207. 10.1038/nbt870 14502203

[B4] Bethea C. L. Lu N. Z. Gundlah C. Streicher J. M. (2002). Diverse actions of ovarian steroids in the serotonin neural system. Front. Neuroendocrinol. 23, 41–100. 10.1006/frne.2001.0225 11906203

[B5] Cairncross K. D. Cox B. Forster C. Wren A. (1977). The ability of local injection of 6-OHDA, 5,6-DHT and 5,7-DHT into the olfactory bulbs to mimic the effects of bilateral bulbectomy in the rat [proceedings]. Br. J. Pharmacol. 61, 145P–146P. 912193 PMC1667625

[B6] Chen J. Condron B. G. (2008). Branch architecture of the fly larval abdominal serotonergic neurons. Dev. Biol. 320, 30–38. 10.1016/j.ydbio.2008.03.038 18561908 PMC2610461

[B7] Deneris E. S. Wyler S. C. (2012). Serotonergic transcriptional networks and potential importance to mental health. Nat. Neurosci. 15, 519–527. 10.1038/nn.3039 22366757 PMC3594782

[B8] Donati R. J. Rasenick M. M. (2003). G protein signaling and the molecular basis of antidepressant action. Life Sci. 73, 1–17. 10.1016/s0024-3205(03)00249-2 12726882

[B9] Goridis C. Rohrer H. (2002). Specification of catecholaminergic and serotonergic neurons. Nat. Rev. Neurosci. 3, 531–541. 10.1038/nrn871 12094209

[B10] Hodges M. R. Tattersall G. J. Harris M. B. McEvoy S. D. Richerson D. N. Deneris E. S.. (2008). Defects in breathing and thermoregulation in mice with near-complete absence of central serotonin neurons. J. Neurosci. 28, 2495–2505. 10.1523/JNEUROSCI.4729-07.2008 18322094 PMC6671195

[B11] Hu B. Y. Weick J. P. Yu J. Ma L. X. Zhang X. Q. Thomson J. A.. (2010). Neural differentiation of human induced pluripotent stem cells follows developmental principles but with variable potency. Proc. Natl. Acad. Sci. U S A 107, 4335–4340. 10.1073/pnas.0910012107 20160098 PMC2840097

[B12] Kelly P. H. Seviour P. W. Iversen S. D. (1975). Amphetamine and apomorphine responses in the rat following 6-OHDA lesions of the nucleus accumbens septi and corpus striatum. Brain Res. 94, 507–522. 1171714 10.1016/0006-8993(75)90233-4

[B13] Kim J. H. Auerbach J. M. Rodriguez-Gomez J. A. Velasco I. Gavin D. Lumelsky N.. (2002). Dopamine neurons derived from embryonic stem cells function in an animal model of Parkinson’s disease. Nature 418, 50–56. 10.1038/nature00900 12077607

[B14] Kumar M. Kaushalya S. K. Gressens P. Maiti S. Mani S. (2009). Optimized derivation and functional characterization of 5-HT neurons from human embryonic stem cells. Stem Cells Dev. 18, 615–627. 10.1089/scd.2008.0181 18800863

[B15] Lee S. H. Lumelsky N. Studer L. Auerbach J. M. McKay R. D. (2000). Efficient generation of midbrain and hindbrain neurons from mouse embryonic stem cells. Nat. Biotechnol. 18, 675–679. 10.1038/76536 10835609

[B16] Liu J. Chu Y. X. Zhang Q. J. Wang S. Feng J. Li Q. (2007). 5,7-dihydroxytryptamine lesion of the dorsal raphe nucleus alters neuronal activity of the subthalamic nucleus in normal and 6-hydroxydopamine-lesioned rats. Brain Res. 1149, 216–222. 10.1016/j.brainres.2007.02.052 17376410

[B17] Lu J. Liu H. Huang C. T. Chen H. Du Z. Liu Y.. (2013). Generation of integration-free and region-specific neural progenitors from primate fibroblasts. Cell Rep. 3, 1580–1591. 10.1016/j.celrep.2013.04.004 23643533 PMC3786191

[B18] Lu J. Zhong X. Liu H. Hao L. Huang C. T. Sherafat M. A.. (2016). Generation of serotonin neurons from human pluripotent stem cells. Nat. Biotechnol. 34, 89–94. 10.1038/nbt.3435 26655496 PMC4711820

[B19] Ma L. Hu B. Liu Y. Vermilyea S. C. Liu H. Gao L.. (2012). Human embryonic stem cell-derived GABA neurons correct locomotion deficits in quinolinic acid-lesioned mice. Cell Stem Cell 10, 455–464. 10.1016/j.stem.2012.01.021 22424902 PMC3322292

[B20] Salli U. Reddy A. P. Salli N. Lu N. Z. Kuo H. C. Pau F. K.. (2004). Serotonin neurons derived from rhesus monkey embryonic stem cells: similarities to CNS serotonin neurons. Exp. Neurol. 188, 351–364. 10.1016/j.expneurol.2004.04.015 15246835

[B21] Takahashi K. Tanabe K. Ohnuki M. Narita M. Ichisaka T. Tomoda K.. (2007). Induction of pluripotent stem cells from adult human fibroblasts by defined factors. Cell 131, 861–872. 10.1016/j.cell.2007.11.019 18035408

[B22] Tokuyama Y. Ingram S. L. Woodward J. S. Bethea C. L. (2010). Functional characterization of rhesus embryonic stem cell-derived serotonin neurons. Exp. Biol. Med. 235, 649–657. 10.1258/ebm.2010.009307 20463306 PMC3031107

[B23] Wichers M. Maes M. (2002). The psychoneuroimmuno-pathophysiology of cytokine-induced depression in humans. Int. J. Neuropsychopharmacol. 5, 375–388. 10.1017/s1461145702003103 12466036

