# Supplementary material for: Characterization of Induced Pluripotent Stem Cell-derived Human Serotonergic Neurons
Source: Front Cell Neurosci. 2017 May 8;11:131. doi: 10.3389/fncel.2017.00131 (PMC5420558; doi:10.3389/fncel.2017.00131)
Supplement: Supplementary file 1 [file Table_1.pdf]

Supplementary Material

Characterization of Induced Pluripotent Stem Cell-derived Human Serotonergic Neurons

Lining Cao[1], Rui Hu[1], Ting Xu[1], Zhen-Ning Zhang[1], Weida Li[1], Jianfeng Lu[1, 2*]

*** Correspondence:** Dr. Jianfeng Lu: LU.JIANFENG@Tongji.edu.cn

Supplementary Table S1: List of antibodies used in this study.

**Name**	**Company**	**Host**	**Dilution**
Serotonin	Abcam	Mouse	1:100
AADC	Chemicon	Rabbit	1:500
GATA2	Sigma	Rabbit	1:500
GATA3	R&D	Mouse	1:1000
GFAP	Dako	Rabbit	1:5000
HTR1a	Millipore	Mouse	1:400
HUMAN NUCLEI	Millipore	Mouse	1:200
Ki67	ZYMED	Rabbit	1:200
MBP	Fisher Scientific	Rabbit	1:500
MHC1	Epitomics	Rabbit	1:200
NeuN	Millipore	Rabbit	1:2000
SERT	ImmunoStar	Rabbit	1:5000
STEM121	Stem cells	Mouse	1:500
TH	Pel-Freez	Rabbit	1:1000
TPH2	Novus Biologicals	Rabbit	1:2000
Tuj1	Covance	Rabbit	1:10000
VMAT2	Abcam	Mouse	1:50
